# Pylephlebitis

**DOI:** 10.4322/acr.2024.473

**Published:** 2024-02-08

**Authors:** Wilker Dias Martins, João Pedro Branco Santana, Marcelo Falcão Barros, Amaro Nunes Duarte

**Affiliations:** 1 Universidade de São Paulo (USP), Faculdade de Medicina, Hospital das Clínicas, Divisão de Anatomia Patológica, São Paulo, SP, Brasil; 2 Universidade de São Paulo (USP), Faculdade de Medicina, Departamento de Patologia, São Paulo, SP, Brasil

**Keywords:** Autopsy, Pathology, Portal Vein

Pylephlebitis is an uncommon entity characterized by septic thrombophlebitis of the portal vein and its tributaries, with more frequent involvement of the right portal vein branch. In 1846, Waller first described it as a possible origin of liver abscesses.^1,2^

The literature on pylephlebitis is restricted to case reports. The estimated incidence is 2.7 cases per 100,000 people per year. It is closely associated with intra-abdominal inflammatory and infectious processes, especially pancreatitis, diverticulitis, and peritonitis. Cholecystitis, as observed in our case, was identified in approximately 7% of pylephlebitis cases. The main risk factors associated with its development are smoking, previous abdominal surgeries, and the use of antiplatelet agents.^2^

Although the pathogenesis of pylephlebitis is not yet well-established, it has been associated with a hypercoagulability state and bacterial translocation, specially by gram-negative bacteria such as *Bacteroides fragilis*, *Escherichia coli*, *Klebsiella pneumoniae*, *Proteus mirabilis* and *Enterobacter spp*.^3^

The clinical picture of pylephlebitis is nonspecific, with fatigue, abdominal pain, fever, nausea, and vomiting, which contributes to a delay in diagnosis and the initiation of therapy, leading to high rates of morbidity and mortality. The typical pathological findings of pylephlebitis are polymorphonuclear inflammatory infiltrate causing venulitis in the portal veins, with endothelial tumefaction, desquamation, and fibrinoid necrosis. The suppurative inflammatory reaction frequently invades the adjacent parenchyma.^4^

Figure 1 refers to the autopsy finding of a 56-year-old man, diagnosed with hypertension, diabetes, and dyslipidemia, who presented with nonspecific abdominal pain, evolving 2 weeks later with fever and loss of appetite. He was admitted to the emergency department with jaundice, septic shock, and encephalopathy, dying one day after admission despite the intensive care treatment. The autopsy revealed an enlarged, congested, and friable liver that weighed 2789 g (reference range: 1500-1800); the gallbladder had a thick wall filled with multiple blackened calculi, compatible with calculous cholecystitis. The histologic exam showed portal venulitis, with fibrino- leukocytic exudate sparing the biliary tract and the hepatic artery associated with numerous hepatic abscesses (Figure 1).

**Figure 1:**
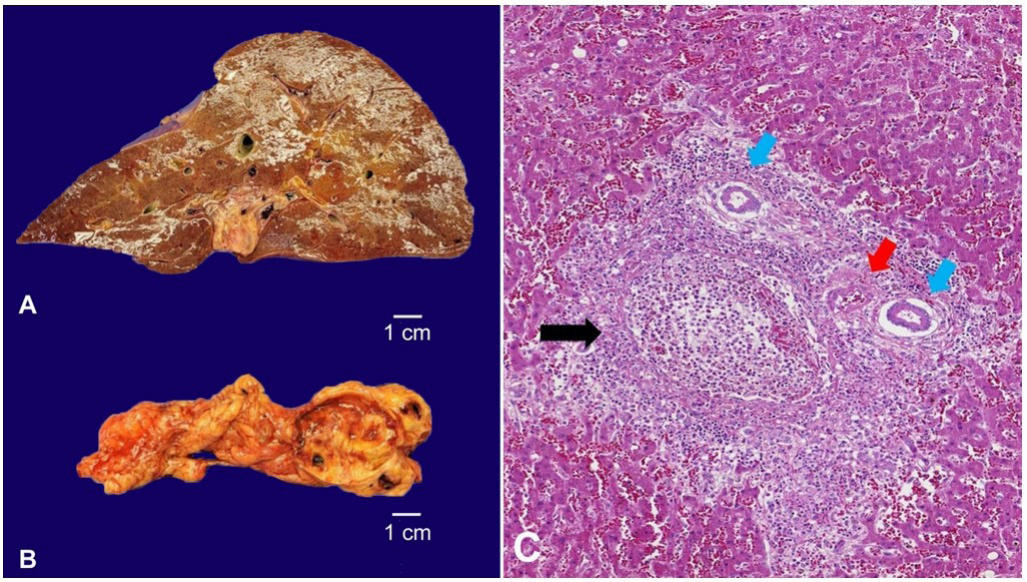
**A -** Gross examination of the congested and friable liver (weight: 2789,0 g); **B -** Gallbladder with chronic inflammation due to calculi; **C -** Photomicrograph of the liver shows portal venulitis, with a suppurative inflammatory reaction and fibrin (black arrow), sparing the biliary tract (blue arrow) and the hepatic artery (red arrow) (H&E, 400X).

## References

[1] Imaoka K, Fukuda S, Tazawa H (2018). A rare case of pylephlebitis as a complication of cholecystocolonic fistula. Case Rep Surg.

[2] Choudhry AJ, Baghdadi YM, Amr MA, Alzghari MJ, Jenkins DH, Zielinski MD (2016). Pylephlebitis: a review of 95 cases. J Gastrointest Surg.

[3] Flores-Anaya L, León-Lozada C, Torres-Damas W (2015). Pylephlebitis: case report and literature review. Medwave.

[4] Fusaro L, Di Bella S, Martingano P, Crocè LS, Giuffrè M (2023). Pylephlebitis: a systematic review on etiology, diagnosis, and treatment of infective portal vein thrombosis. Diagnostics.

